# DCJ-indel and DCJ-substitution distances with distinct operation costs

**DOI:** 10.1186/1748-7188-8-21

**Published:** 2013-07-23

**Authors:** Poly H da Silva, Raphael Machado, Simone Dantas, Marília DV Braga

**Affiliations:** 1 IME, Universidade Federal Fluminense, Niterói, Brazil; 2 Inmetro – Instituto Nacional de Metrologia, Qualidade e Tecnologia, Duque de Caxias, Brazil

**Keywords:** Double cut and join (DCJ), Insertions and deletions (indels), Substitution, Genome rearrangements, Genomic distance, Evolution, Comparative genomics, Combinatorics, Algorithms

## Abstract

**Background:**

Classical approaches to compute the genomic distance are usually limited to genomes with the same content and take into consideration only rearrangements that change the organization of the genome (i.e. positions and orientation of pieces of DNA, number and type of chromosomes, etc.), such as inversions, translocations, fusions and fissions. These operations are generically represented by the double-cut and join (DCJ) operation. The distance between two genomes, in terms of number of DCJ operations, can be computed in linear time. In order to handle genomes with distinct contents, also insertions and deletions of fragments of DNA – named *indels* – must be allowed. More powerful than an indel is a *substitution* of a fragment of DNA by another fragment of DNA. Indels and substitutions are called *content-modifying* operations. It has been shown that both the DCJ-indel and the DCJ-substitution distances can also be computed in linear time, assuming that the same cost is assigned to any DCJ or content-modifying operation.

**Results:**

In the present study we extend the DCJ-indel and the DCJ-substitution models, considering that the content-modifying cost is distinct from and upper bounded by the DCJ cost, and show that the distance in both models can still be computed in linear time. Although the triangular inequality can be disrupted in both models, we also show how to efficiently fix this problem *a posteriori*.

## Background

The distance between two genomes is often computed using only the common markers, that occur in both genomes. Such distance allows rearrangements that change the organization of the genome, that is, the positions and orientations of markers, number and types of chromosomes. Inversions, translocations, fusions and fissions are some of these operations [1]. All these rearrangements can be generically represented as a *double-cut-and-join* (DCJ) operation [2]. The DCJ distance, which takes into consideration only DCJ operations, can be computed in linear time [3].

Nevertheless, genomes with the same content are rare, and differences in gene content may reflect important evolutionary aspects. In order to handle genomes with unequal contents, one has to take into consideration *content-modifying* operations, that change the contents of the genomes. These operations can be an *insertion* or a *deletion* of a piece of DNA. Insertions and deletions are also called *indels*. Some extensions of the classical approaches lead to models that handle genomes with unequal contents, but without duplicated markers, allowing rearrangements and indels. In 2001, El Mabrouk [4] extended the classical sorting by inversions approach [5] and developed a method to compare unichromosomal genomes with unequal contents, considering only inversions and indels. She provided an exact algorithm that deals with insertions and deletions asymmetrically, and a heuristic that handles the operations symmetrically. Then, in 2009, a model to sort multichromosomal genomes with unequal contents, using both DCJ and indel operations was introduced by Yancopoulos and Friedberg [6]. Later, Braga *et al.*[7] presented an exact formula for the DCJ-indel distance, that can be computed in linear time handling indels symmetrically.

Recently, in 2011, a more powerful content-modifying operation has also been considered: a *substitution* allows a piece of DNA to be substituted by another piece of DNA [8]. Observe that it is not suggested that a substitution occurs in a precise moment in evolution, but instead it represents a region that underwent continuous mutations (duplications, losses and gene mutations), so that a group of genes is transformed into a different group of genes (either of which may also be empty, allowing a substitution to represent an insertion or a deletion). Other studies also represent continuous mutations as a rearrangement event [9,10]. By minimizing substitutions we are able to establish a relation between indels that could have occurred in the same position of the compared genomes, identifying genomic regions that could be subject to these continuous mutations. It has been shown that the DCJ-substitution distance can also be computed in linear time [8].

The approaches mentioned above [4,6-8] assign the same cost to any rearrangement or content-modifying operation. However, during the evolution of many organisms, content-modifying operations are said to occur more often than rearrangements and, consequently, should be assigned to a lower cost. Examples are bacteria that are obligate intracellular parasites, such as *Rickettsia*[11]. The genomes of such intracellular parasites are observed to have a reductive evolution, that is, the process by which genomes shrink and undergo extreme levels of gene degradation and loss. In the present work, we refine the DCJ-indel [7] and the DCJ-substitution [8] models, by adopting a distinct content-modifying cost that is upper bounded by the DCJ cost. For simplicity, we assign a cost of 1 to DCJ and a positive cost of *w*≤1 to content-modifying operations. We are then able to give exact formulas for both the DCJ-indel and the DCJ-substitution distances, for any positive *w*≤1.

Content-modifying operations are applied to pieces of DNA of any size, and a side effect of this fact is that the triangular inequality often does not hold for distances that consider these operations [4,6-8,12]. In the case of the models we study here, it is possible to do an *a posteriori* correction, using an approach similar to the one described in [12].

This paper is an extension of [13] and is organized as follows. In the remainder of this section we give definitions and previous results used in this work. We will then present our results, including the formulas for the distances with distinct DCJ and content-modifying costs and the correction to establish the triangular inequality.

### Genomes

We deal with models in which duplicated markers are not allowed. Given two genomes *A* and *B*, possibly with unequal content, let G, A and B be three disjoint sets, such that G is the set of markers that occur both in *A* and *B*, A is the set of markers that occur only in *A*, and B is the set of markers that occur only in *B*. The markers in sets A and B are also called *unique markers*. We denote by u(A,B)=|A|+|B| the number of unique markers in genomes *A* and *B*.

**Figure 1:**
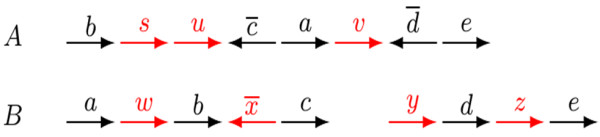
**Genomes **$A=\left\{\circ \text{bsu}\bar{c}\text{av}\bar{d}e\circ \right\}$**, composed of one single linear chromosome, and**$B=\{\circ \text{awb}\bar{x}c\circ$**, ∘*ydze*∘}, composed of two linear chromosomes.** The markers in G are represented in black, while the unique markers in A and in B are represented in red.

Each marker *g* in a genome is a DNA fragment and is represented by the symbol *g*, if it is read in direct orientation, or by the symbol $\bar{g}$, if it is read in reverse orientation. Each one of the two extremities of a linear chromosome is called a *telomere*, represented by the symbol ∘. Each chromosome in a genome can be then represented by a string that can be circular, if the chromosome is circular, or linear and flanked by the symbols ∘ if the chromosome is linear. In general, a genome is either circular (composed of circular chromosomes) or linear (composed of linear chromosomes). As an example, consider the linear genomes $A=\left\{\circ \text{bsu}\bar{c}\text{av}\bar{d}e\circ \right\}$ and $B=\left\{\circ \text{awb}\bar{x}c\circ ,\circ \text{ydze}\circ \right\}$, represented in Figure 1. Here we have G={a,b,c,d,e}, A={s,u,v} and B={w,x,y,z}.

### The DCJ model

In this section we will summarize the DCJ model, that allows the sorting of the common content of two genomes, also called *DCJ-sorting*. We will also show how the DCJ distance can be easily computed with the help of the *adjacency graph*.

Given two genomes *A* and *B*, we denote the two extremities of each g∈G by *g*^*t*^ (tail) and *g*^*h*^ (head). Then, a G*-adjacency* or simply *adjacency*[7] in genome *A* (respectively in genome *B*) is a string $v=\gamma_{1}\ell \gamma_{2}\equiv \gamma_{2}\bar{\ell }\gamma_{1}$, such that each *γ*_*i*_ can be a telomere or an extremity of a marker from G and *ℓ* is a substring composed of the markers that are between *γ*_1_ and *γ*_2_ in *A* (respectively in *B*) and contains no marker that also belongs to G. The substring *ℓ* is the *label* of *v*. If *ℓ* is empty, the adjacency is said to be *clean*, otherwise it is said to be *labeled*. If a linear chromosome is composed only of unique markers, it is represented by an adjacency ∘*ℓ*∘. Similarly, a circular chromosome composed only of unique markers is represented by a (circular) adjacency *ℓ*. For the linear genomes represented in Figure 1, the set of adjacencies in *A* is {∘*b*^*t*^, *b*^*h*^*suc*^*h*^, *c*^*t*^*a*^*t*^, *a*^*h*^*vd*^*h*^, *d*^*t*^*e*^*t*^, *e*^*h*^ ∘} and the set of adjacencies in *B* is $\left\{\circ a^{t},\;a^{h}\;wb^{t},\;b^{h}\bar{x}c^{t},\;c^{h}\;\circ ,\;\circ yd^{t},\;d^{h}\;ze^{t},\;e^{h}\;\circ \right\}$.

#### Adjacency graph

**Figure 2:**
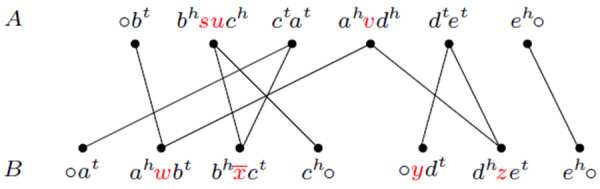
**For genomes *A *and *B *(Figure 1), the graph has one *BB *and two *AB*-paths**

Given two genomes *A* and *B*, the *adjacency graphAG*(*A*, *B*) [3] is the bipartite multigraph whose vertices are the adjacencies of *A* and of *B* and that has one edge for each common extremity of a pair of vertices. Each of the connected components of *AG*(*A*, *B*) alternate vertices in genome *A* and in genome *B*. Each component can be either a cycle, or an *AB-path* (that has one endpoint in genome *A* and the other in *B*), or an *AA-path* (that has both endpoints in genome *A*), or a *BB-path* (that has both endpoints in *B*). A special case of an *AA* or a *BB*-path is a *linear singleton*, that is a linear chromosome represented by an adjacency of type ∘*ℓ*∘, where *ℓ* contains only unique markers. Paths occur when the genomes are linear. For circular genomes, the graph *AG*(*A*, *B*) is composed of cycles only, and may also have a special type of component composed of a single vertex, that corresponds to a circular chromosome composed only of markers that are not in G, called *circular singleton*. In Figure 2 we show the adjacency graph built over the linear genomes represented in Figure 1.

#### DCJ operations

A *cut* performed on a genome *A* separates two adjacent markers of *A*. A cut affects a single adjacency *v* in *A*: it is done between two symbols of *v*, creating two open ends. In general a cut can be performed between two markers of a label, but the DCJ-indel distance can be computed considering only cuts that do not “break” labels. A *double-cut and join* or *DCJ* applied on a genome *A* is the operation that performs cuts in two different adjacencies in *A*, creating four open ends, and joins these open ends in a different way. In other words, a DCJ rearranges two adjacencies in *A*, transforming them into two new adjacencies. As an example consider a DCJ applied to genome *A* (from Figure 1), that rearranges the adjacencies *a*^*h*^*vd*^*h*^ and *d*^*t*^*e*^*t*^ into the new adjacencies *a*^*h*^*vd*^*t*^ and *d*^*h*^*e*^*t*^. Observe that this operation corresponds to the inversion of marker *d* in genome *A*. Indeed, a DCJ operation can correspond to several rearrangements, such as an inversion, a translocation, a fusion or a fission [2].

#### DCJ-sorting and DCJ distance

Given two genomes *A* and *B*, the components of *AG*(*A*, *B*) with 3 or more vertices need to be reduced, by applying DCJ operations, to components with only 2 vertices, that can be cycles or *AB*-paths [14]. This procedure is called *DCJ-sorting* of *A* into *B*. The number of *AB*-paths in *AG*(*A*, *B*) is always even and a DCJ can be of three types [7]: it can either decrease the number of cycles by one, or the number of *AB*-paths by two (*counter-optimal*); or it does not affect the number of cycles and *AB*-paths (*neutral*); or it can either increase the number of cycles by one, or the number of *AB*-paths by two (*optimal*). The DCJ distance of *A* and *B*, denoted by *d*_*DCJ*_(*A*, *B*), is the minimum number of steps required to do a DCJ-sorting of *A* into *B*, given by the following theorem.

**Theorem 1** (from [3]). *Given two genomes A and B, we have*$d_{\;\text{DCJ}}(A,\;B)=|G|-c-\frac{b}{2}$*, where*G* is the set of common markers and c and b are, respectively, the number of cycles and of AB-paths in AG*(*A*, *B*).

#### Internal DCJ operations and recombinations

Observe that a DCJ operation *ρ* acts on two different adjacencies, that can be in the same or in two distinct connected components of the graph. The components on which the cuts are applied are called *sources* and the components obtained after the joinings are called *resultants* of *ρ*. With respect to the adjacency graph, *ρ* can be of two types: *internal*, when *ρ* is applied to two adjacencies belonging to a single component; and *recombination*, when *ρ* is applied to adjacencies belonging to two distinct components.

Any recombination applied to a vertex of an *AA*-path and a vertex of a *BB*-path is optimal [14]. A recombination applied to vertices of two distinct *AB*-paths can be either neutral, when the resultants are also *AB*-paths, or counter-optimal, when the resultants are an *AA*-path and a *BB*-path. All other types of path recombinations are neutral and all recombinations involving at least one cycle are counter-optimal.

It is possible to do a separate DCJ-sorting in any component *P* of *AG*(*A*, *B*) [14] by applying DCJs internal to *P*. We denote by *d*_*DCJ*_(*P*) the number of optimal DCJ operations used for DCJ-sorting *P* separately (*d*_*DCJ*_(*P*) depends only on the number of vertices or, equivalently, the number of edges of *P*[14]). Thus, the DCJ distance can also be re-written in terms of the sum of the distance per component:

**Lemma 1** (derived from [14]). *Given two genomes A and B, we have*$d_{\;\text{DCJ}}(A,\;B)=\underset{P\in A\;G(A,\;B)}{\sum }d_{\;\text{DCJ}}(P)$.

Only optimal DCJs, counted in the equivalent formulas given by Theorem 1 and Lemma 1, are necessary to do a DCJ-sorting. Given a DCJ *ρ*, the *DCJ variation* of *ρ*, denoted by *Δ*_*DCJ*_(*ρ*), is defined to be respectively 0, 1 and 2 depending whether *ρ* is optimal, neutral or counter-optimal.

### Modifying the content of a genome

In the previous section, the unique markers appeared as labels of adjacencies, but the DCJ operations are only able to change the organization of the genomes. Here we introduce the operations that are applied to the labels and change the content of the genomes.

#### Indel operations

The most classical content-modifying operations are *insertions* and *deletions* of blocks of contiguous markers [4,6]. We refer to insertions and deletions as *indel* operations. In the model we consider, an indel only affects the label of one single adjacency, by deleting or inserting contiguous markers in this label, with the restriction that an insertion cannot produce duplicated markers [7]. Thus, while sorting *A* into *B*, the indels are the steps in which the markers in A are deleted and the markers in B are inserted. At most one chromosome can be entirely deleted or inserted at once. We illustrate an indel with the following example: the deletion of markers *su* from adjacency *b*^*h*^*suc*^*h*^ of genome *A* (Figure 2), which results into the clean adjacency *b*^*h*^*c*^*h*^. The opposite operation would be an insertion.

#### Substitutions

*Substitutions* are more powerful content-modifying operations, that allow blocks of contiguous markers to be substituted by other blocks of contiguous markers [8]. In other words, a deletion and a subsequent insertion that occur at the same position of the genome can be modeled as a substitution, counting together for one single sorting step.

A substitution only affects the label of one single adjacency, by substituting contiguous markers in this label, with the restriction that it cannot produce duplicated markers [8]. An example is the substitution of markers *su* in adjacency *b*^*h*^*suc*^*h*^ by $\bar{x}$, which results into adjacency $b^{h}\bar{x}c^{h}$. At most one chromosome can be entirely substituted at once (but we do not allow the substitution of a linear by a circular chromosome nor *vice-versa*). As previously mentioned, insertions and deletions are special cases of substitutions. If a block of markers is substituted by the empty string, we have a deletion. Analogously, if the empty string is substituted by a block of markers, we have an insertion.

### Runs, indel- and substitution-potentials

In this section we introduce some definitions and concepts that will help us to integrate the DCJ model with content-modifying operations. These concepts will be very useful in our results, when we will show how to use DCJ operations to minimize the number of content-modifying operations to be performed.

**Figure 3:**
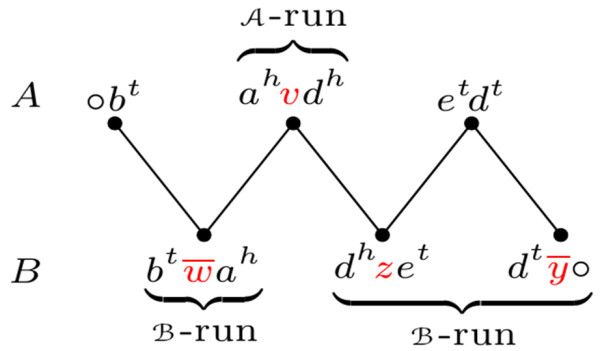
**An *AB*-path with 3 runs (extracted from Figure 2).**

First, let us recall the concept of *run*, introduced in [7]. Given two genomes *A* and *B* and a component *P* of *AG*(*A*, *B*), a *run* is a maximal subpath of *P*, in which the first and the last vertices are labeled and all labeled vertices belong to the same genome (or partition). An example is given in Figure 3. A run in genome *A* is also called an A-run, and a run in genome *B* is called a B-run. We denote by *Λ*(*P*) the number of runs in a component *P*. While a path can have any number or runs, a cycle has either 0, 1, or an even number of runs.

A set of labels of one genome can be accumulated with DCJs. For example, take the adjacencies *d*^*h*^*ze*^*t*^ and $d^{t}\;\bar{y}\circ$ from genome *B* (Figure 3). A DCJ applied to these two adjacencies could result into *d*^*t*^*e*^*t*^ and $d^{h}\;z\bar{y}\circ$, in which the label $z\bar{y}$ resulted from the accumulation of the labels of the two original adjacencies. In particular, when we apply optimal DCJs internal to a single component of the adjacency graph, we can accumulate an entire run into a single adjacency [7].

**Figure 4:**
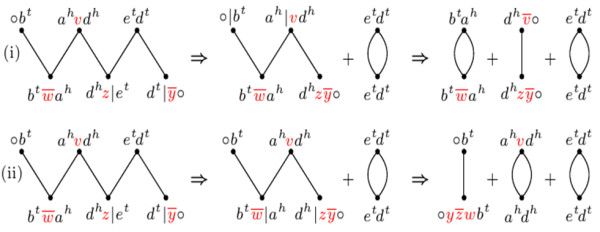
**Two optimal sequences for DCJ-sorting an *AB*-path with *Λ * =3 (the cuts of each DCJ in each sequence are represented by “ |”).** In **(i)** the overall number of runs in the resulting components is three, while in **(ii)** the resulting components have only two runs. Indeed, in this case, the best we can have is the indel-potential *λ*=2.

Runs can be merged by DCJ operations. Consequently, during the optimal DCJ-sorting of a component *P*, we can reduce its number of runs. The *indel-potential* of *P*, denoted by *λ*(*P*), is defined in [7] as the minimum number of runs that we can obtain by DCJ-sorting *P* with optimal DCJ operations. An example is given in Figure 4.

The indel-potential of a component depends only on its number of runs:

**Proposition 1** (from [7]).*Given two genomes A and Band a component P of AG*(*A*, *B*),* the indel-potential of P is given by*$\lambda (P)=\lceil \frac{\Lambda (P)+1}{2}\rceil$*, if Λ*(*P*)≥1. *Otherwise, if Λ*(*P*)=0, *then λ*(*P*)=0.

Similarly, the *substitution-potential* of a component *P* is the minimum number of substitutions that we can obtain by DCJ-sorting *P* with optimal DCJ operations. The substitution-potential is denoted by *σ*(*P*) and can be computed as follows:

**Proposition 2** (from [8]). *Given genomes A and B and a component P* of *AG*(*A*, *B*), *the substitution-potential of P is given by*$\sigma (P)=\lceil \frac{\Lambda (P)+1}{4}\rceil$, *if Λ*(*P*)≥1. *Otherwise, if Λ*(*P*)=0, *then σ*(*P*)=0.

## Results

In this section we show how to compute the DCJ-indel and the DCJ-substitution distances, considering that the content-modifying cost is distinct from and upper bounded by the DCJ cost. We assign the cost of 1 to each DCJ and a positive cost *w*≤1 to each content-modifying operation.

### The DCJ-indel model with distinct operation costs

First we consider the case in which only indels are allowed as content-modifying operations. Given two genomes *A* and *B*, we define the *DCJ-indel distance* of *A* and *B*, denoted by $d_{\text{DCJ}}^{\text{id}}(A,B)$, as the minimum cost of a DCJ-indel sequence of operations that sorts *A* into *B*. If *w*=1, the DCJ-indel distance corresponds exactly to the minimum number of steps required to sort *A* into *B*. To compute the distance in this case, a linear algorithm was given in [7]. Here we present a more general method to compute the DCJ-indel distance for any positive *w*≤1.

#### An upper bound for the DCJ-indel distance

We can obtain a good upper bound for the DCJ-indel distance by showing how to compute the DCJ-indel distance per component. Given a DCJ operation *ρ*, let *λ*_0_ and *λ*_1_ be, respectively, the sum of the indel-potentials for the components of the adjacency graph before and after *ρ*, and let *Δλ*(*ρ*)=*λ*_1_−*λ*_0_. If *ρ* is an optimal DCJ internal to a single component of the graph, the definition of indelpotential implies *Δλ*(*ρ*)≥0. We also have *Δλ*(*ρ*)≥0, if *ρ* is counter-optimal, and *Δλ*(*ρ*)≥−1, if *ρ* is neutral [7]. Recall that *Δ*_*DCJ*_(*ρ*) is, respectively, 0, 1 and 2, depending whether the DCJ *ρ* is optimal, neutral or counter-optimal. We define *Δ*_*DCJ*-*λ*_(*ρ*)=*Δ*_*DCJ*_(*ρ*) + *wΔλ*(*ρ*).

We know that each component *P* of *AG*(*A*,*B*) can be DCJ-sorted separately, and the labels can then be easily sorted with indel operations. Let $d_{\text{DCJ}}^{\text{id}}(P)$ be the DCJ-indel distance of *P*, that is the minimum cost of a DCJ-indel sequence of operations sorting *P* separately. This can be computed according to the following proposition.

**Proposition 3. ***For each P*∈*AG*(*A*, *B*), $d_{\text{DCJ}}^{\text{id}}(P)=d_{\text{DCJ}}(P)+\mathrm{w\lambda}(P)$.

*Proof.* By the definition of *λ*, the best we can do with optimal DCJs is *d*_*DCJ*_(*P*)+*wλ*(*P*). From [7], we have *Δ*_*DCJ*-*λ*_(*ρ*)≥2 if *ρ* is counter-optimal, thus we can only get more expensive sorting scenarios if we use such operation. We also know that, if *ρ* is neutral *Δ*_*DCJ*-*λ*_(*ρ*)≥1−*w*≥0, for any positive *w*≤1. □

This allows us to get a good upper bound for the DCJ-indel distance with distinct operation costs:

**Lemma 2.***Given two genomes A and B and a positive indel costw*≤1, *we have*

$$d_{\text{DCJ}}^{\text{id}}(A,\;B)\leq d_{\text{DCJ}}(A,B)+w\;\underset{P\in A\;G(A,B)}{\sum }\;\lambda (P).$$

*Proof.* If we sort the components separately we have $d_{\text{DCJ}}^{\text{id}}(A,B)\leq \underset{P\in A\;G(A,\;B)}{\sum }d_{\text{DCJ}}^{\text{id}}(P)$, which, according to Lemma 1 and Proposition 3, corresponds exactly to $d_{\text{DCJ}}(A,B)+w\underset{P\in A\;G(A,B)}{\sum }\lambda (P)$.

#### Recombinations and the exact DCJ-indel distance

Until this point, we have explored the possible effects of any DCJ that is internal to a single component of the graph. Now we will analyze the effect of recombinations, that have *Δλ*≥−2 [7]. We saw previously that any recombination involving cycles is counter-optimal. Since any counter-optimal recombination has *Δ*_*DCJ*-*λ*_≥2−2*w*≥0, only path recombinations can have *Δ*_*DCJ*-*λ*_ <0.

Although the space of recombinations is not small, some observations allow us to explore it efficiently. Proposition 1 shows that the indel-potential increases of one when the number of runs increases of two. Furthermore, when we decrease the number of runs of a path by one, it will decrease the indel-potential only if its initial number of runs is one or a multiple of two. However, the exact number of runs does not really matter. In the path recombination analysis, we only have to consider the following properties for each path: 

•whether it is an *AA*, or a *BB*, or an *AB*-path;

•whether it has zero, or an odd or an even number of runs; and

•whether its first run is in *A* or in *B* (by convention, an *AB*-path is always read from *A* to *B*).

**Figure 5:**
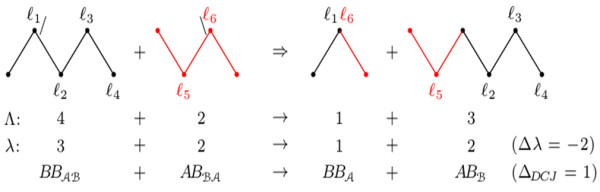
**Neutral recombination that has *Δ***_***DCJ*-*λ***_**=1−2*w* (we represent only the labels of the adjacencies, the cuts of the recombination are represented by “ /” and “ ∖”).**

An empty sequence (with no run) is represented by *ε*. For the benefit of the reader, for an integer *i*≥0, let A (respectively B) be a sequence with odd 2*i*+1 runs, starting and ending with an A-run (respectively B-run). Similarly, let AB (respectively BA), be a sequence with even 2*i*+2 runs, starting with an A-run (respectively B-run) and ending with a B-run (respectively A-run). Then each one of the notations *AA*_*ε*_, $AA_{A}$, $AA_{B}$, $AA_{AB}\equiv AA_{BA}$, *BB*_*ε*_, $BB_{A}$, $BB_{B}$, $BB_{AB}\equiv BB_{BA}$, *AB*_*ε*_, $AB_{A}$, $AB_{B}$, $AB_{AB}$ and $AB_{BA}$ represents a particular type of path (*AA*, *BB* or *AB*) with a particular structure of runs (*ε*, A, B, AB or BA). An example of this notation is given in Figure 5, which represents a neutral recombination possibly with *Δ*_*DCJ*-*λ*_ <0.

Each type of recombination can lead to different resultants, depending on where the cuts are applied. However, it is always possible to choose the “best” resultants in each case: we take the recombination with the smallest *Δ*+_*DCJ*-*λ*_, whose resultants can be better reused in further recombinations. The main observations to guide this task are: only recombinations of paths whose runs are AB or BA have *Δλ*=−2 and only recombinations of type *AA*+*BB* are optimal and have *Δ*_*DCJ*_=0. In Table 1, we list all path recombinations that can have *Δ*_*DCJ*-*λ*_<0, together with neutral recombinations that have *Δ*_*DCJ*-*λ*_=1−*w*≥0, but produce an $AA_{AB}$ or a $BB_{AB}$ path. We denote by ∙ an *AB*-path that never appears as a source of a recombination in this table (these paths are *AB*_*ε*_, $AB_{A}$ and $A\;B_{B}$).

**Table 1:** Path recombinations that are used to compute the DCJ-indel distance

	**Sources**	**Resultants**	***Δλ***	***Δ***_***DCJ***_	***Δ***_***DCJ***-***λ***_		**Sources**	**Resultants**	***Δλ***	***Δ***_***DCJ***_	***Δ***_***DCJ-λ***_
*o*_-2_	$AA_{A\;B}+BB_{AB}$	∙+∙	−2	0	−2*w*						
						*n*_-2_	$AA_{AB}+AA_{AB}$	$AA_{A}+AA_{B}$	−2	1	1−2*w*
*o*_-1_	$\;\;AA_{A}+BB_{AB}$	$∙+AB_{AB}$	−1	0	−*w*	*n*_-2_	$BB_{AB}+BB_{AB}$	$\;BB_{A}+BB_{B}$	−2	1	1−2*w*
*o*_-1_	$\;\;BB_{A}+AA_{AB}$	$∙+AB_{BA}$	−1	0	−*w*	*n*_-2_	$AA_{AB}+AB_{AB}$	$\;\;\;\;\;\;\;∙+AA_{A}$	−2	1	1−2*w*
*o*_-1_	$\;\;AA_{B}+BB_{AB}$	$∙+AB_{BA}$	−1	0	−*w*	*n*_-2_	$AA_{AB}+AB_{BA}$	$\;\;\;\;\;\;\;∙+AA_{B}$	−2	1	1−2*w*
*o*_-1_	$\;\;BB_{B}+AA_{AB}$	$∙+AB_{AB}$	−1	0	−*w*	*n*_-2_	$BB_{AB}+AB_{AB}$	$\;\;\;\;\;\;\;∙+BB_{B}$	−2	1	1−2*w*
*o*_-1_	$AA_{A}+BB_{A}$	∙+∙	−1	0	−*w*	*n*_-2_	$BB_{AB}+AB_{BA}$	$\;\;\;\;\;\;\;∙+BB_{A}$	−2	1	1−2*w*
*o*_-1_	$AA_{B}+BB_{B}$	∙+∙	−1	0	−*w*	*n*_-2_	$AB_{AB}+AB_{BA}$	∙+∙	−2	1	1−2*w*
*n*_-1_	$AA_{A}+AB_{BA}$	$∙+AA_{AB}$	−1	1	1−*w*	*n*_-1_	$BB_{A}+AB_{AB}$	$\;\;\;\;\;\;\;∙+BB_{AB}$	−1	1	1−*w*
*n*_-1_	$AA_{B}+AB_{AB}$	$∙+AA_{AB}$	−1	1	1−*w*	*n*_-1_	$BB_{B}+AB_{BA}$	$\;\;\;\;\;\;\;∙+BB_{AB}$	−1	1	1−*w*

Recombinations of type ***o***_**-*2***_ (optimal with ***Δλ=−2***), ***o***_**-*1***_ (optimal with ***Δλ=−1***) and ***n***_**-*2***_ (neutral with ***Δλ=−2***) can have ***Δ***_***DCJ*-*λ***_***<0***. Recombinations of type ***n***_**-*1***_ (neutral with ***Δλ=−1***) have ***Δ***_***DCJ*-*λ***_***=1−w≥0***, but produce an ${\mathrm{AA}}_{AB}$ or a ${\mathrm{BB}}_{AB}$ path.

##### The DCJ-indel distance formula

By analyzing the whole universe of operations, we could identify groups of recombinations, as listed in Table 2. Since some resultants of recombinations can be used in other recombinations, the groups can have more than one recombination. Groups P, $S_{1}$ and $S_{2}$ are composed of a single recombination, while groups T, $N_{1}$ and $N_{2}$ are composed of two recombinations and groups Q and M are composed of three recombinations. recombination is not an associative operation, thus, in column ‘DCJ seq.’ of Table 2, we indicate how the sequence of DCJs must be applied in each group (the symbol ≺ separates preceeding and succeeding recombinations).

**Table 2:** All recombination groups that determine the deductions for computing the DCJ-indel distance

	**Sources**	**Resultants**	**DCJ seq.**	***Δ***_***DCJ*-*λ***_	**skip if**
P	$AA_{AB}+BB_{AB}$	2 ∙	*o*_-2_	−2*w*	
Q	$2AA_{AB}+BB_{A}+BB_{B}$	4 ∙	2*o*_-1_≺*n*_-2_	1−4*w*	$w\leq \frac{1}{2}$
	$2BB_{AB}+AA_{A}+AA_{B}$	4 ∙		1−4*w*	
T	$AA_{AB}+BB_{A}+AB_{AB}$	3 ∙	*o*_-1_≺*n*_-2_	1−3*w*	$w\leq \frac{1}{2}$
	$AA_{AB}+BB_{B}+AB_{BA}$	3 ∙		1−3*w*	
	$BB_{AB}+AA_{A}+AB_{BA}$	3 ∙		1−3*w*	
	$BB_{AB}+AA_{B}+AB_{AB}$	3 ∙		1−3*w*	
	$2BB_{AB}+AA_{A}$	$2\;∙+BB_{B}$		1−3*w*	
	$2BB_{AB}+AA_{B}$	$2\;∙+BB_{A}$		1−3*w*	
	$2AA_{AB}+BB_{A}$	$2\;∙+AA_{B}$		1−3*w*	
	$2AA_{AB}+BB_{B}$	$2\;∙+AA_{A}$		1−3*w*	
$S_{1}$	$AA_{A}+BB_{A}$	2 ∙	*o*_-1_	−*w*	
	$AA_{B}+BB_{B}$	2 ∙		−*w*	
	$AA_{AB}+BB_{A}$	$∙+AB_{BA}$		−*w*	
	$AA_{AB}+BB_{B}$	$∙+AB_{AB}$		−*w*	
	$BB_{AB}+AA_{A}$	$∙+AB_{AB}$		−*w*	
	$BB_{AB}+AA_{B}$	$∙+AB_{BA}$		−*w*	
$S_{2}$	$AB_{AB}+AB_{BA}$	2 ∙	*n*_-2_	1−2*w*	$w\leq \frac{1}{2}$
	$AA_{AB}+AB_{AB}$	$∙+AA_{A}$		1−2*w*	
	$AA_{AB}+AB_{BA}$	$∙+AA_{B}$		1−2*w*	
	$BB_{AB}+AB_{AB}$	$∙+BB_{B}$		1−2*w*	
	$BB_{AB}+AB_{BA}$	$∙+BB_{A}$		1−2*w*	
	$AA_{AB}+AA_{AB}$	$AA_{A}+AA_{B}$		1−2*w*	
	$BB_{AB}+BB_{AB}$	$BB_{A}+BB_{B}$		1−2*w*	
M	$2AB_{AB}+AA_{B}+BB_{A}$	4 ∙	2*n*_-1_≺*o*_-2_	2−4*w*	$w\leq \frac{1}{2}$
	$2AB_{BA}+AA_{A}+BB_{B}$	4 ∙		2−4*w*	
$N_{1}$	$AB_{AB}+AA_{B}+BB_{A}$	$2\;∙+AB_{BA}$	*n*_-1_≺*o*_-1_	1−2*w*	$w\leq \frac{1}{2}$
	$AB_{BA}+AA_{A}+BB_{B}$	$2\;∙+AB_{AB}$		1−2*w*	
$N_{2}$	$2AB_{AB}+AA_{B}$	$2\;∙+AA_{A}$	*n*_-1_≺*n*_-2_	2−3*w*	$w\leq \frac{2}{3}$
	$2AB_{AB}+BB_{A}$	$2\;∙+BB_{B}$		2−3*w*	
	$2AB_{BA}+AA_{A}$	$2\;∙+AA_{B}$		2−3*w*	
	$2AB_{BA}+BB_{B}$	$2\;∙+BB_{A}$		2−3*w*	

While in groups Q and T the preceding recombinations have lower *Δ*_*DCJ*-*λ*_, in groups M, $N_{1}$ and $N_{2}$ we need to use operations of type *n*_-1_ in order to prepare better recombinations. Another important observation concerning groups Q and T is that, although their *Δ*_*DCJ*-*λ*_ indicate that Q could be applied for *w*>1/4 and T could be applied for *w*>1/3, the last operation of these groups is of type *n*_-2_ and actually increases *Δ*_*DCJ*-*λ*_ for *w*≤1/2. For this reason, we skip groups Q and T for *w*≤1/2 (there is no loss with this approach, since their optimal operations are then counted in $S_{1}$).

The deductions shown in Table 2 can be computed with an approach that greedily maximizes the number of occurrences in P, Q, T, $S_{1}$, $S_{2}$, M, $N_{1}$ and $N_{2}$ in this order. The two groups in Q are mutually exclusive after maximizing P. The lines in T are subgroups of the lines in Q, that is, they are only computed when there are enough remaining components after maximizing Q. Similarly, each one of the remaining groups are computed when there are enough remaining components after maximizing the upper groups. With the results presented in this section we have an exact formula to compute the DCJ-indel distance:

**Theorem 2.** Given two genomes A and B and a positive indel cost *w*≤1,

$$\begin{array}{lll} d_{\text{DCJ}}^{\text{id}}(A,B) & =d_{\text{DCJ}}(A,B)+w\underset{P\in A\;G(A,\;B)}{\sum }\lambda (P)-\;2wP\; & \;\\ & \;-(4w-1)Q-(3w-1)T\; & \;\\ & \;-\;wS_{1}-(2w-1)(S_{2}+2M+N_{1})\; & \;\\ & \;-\;(3w-2)N_{2},\; & \; \end{array}$$

where P, Q, T, $S_{1}$, $S_{2}$, M, $N_{1}$ and $N_{2}$ are computed as described above.

As we mentioned before, the groups Q and T are skipped ( Q=T=0) for *w*≤1/2. Furthermore, we also have $S_{2}=M=N_{1}=0$ if *w*≤1/2 and $N_{2}=0$ if *w*≤2/3. Although some groups have reusable resultants, those are actually never reused (if groups that are lower in the table use as sources resultants from higher groups, the sources of all referred groups would be previously consumed in groups that occupy even higher positions in the table). Due to this fact, the number of occurrences in each group depends only on *w* and the initial number of each type of component.

Observe that, for *w*=1, our formula is identical to the one proposed in [7]. Actually, for any 2/3<*w*≤1, the two formulas are equivalent, since the same occurrences of groups of recombinations and an equivalent upper bound are taken into account.

We illustrate the result of our formula with an example. Let *AG*(*A*,*B*) have only the following labeled paths: two $AA_{AB}$, one $BB_{A}$ and one $BB_{B}$. In this case, there are no occurrences of P, thus we have P=0. If we take $w>\frac{1}{2}$, all labeled paths are consumed in one occurrence of Q. We have Q=1, while all other values are zero, resulting in *Δ*_*DCJ*-*λ*_=1−4*w*. On the other hand, if $w\leq \frac{1}{2}$, we automatically set $Q=T=S_{2}=M=N_{1}=N_{2}=0$. The labeled paths are consumed in two occurrences of $S_{1}$, that is, $S_{1}=2$, resulting in *Δ*_*DCJ*-*λ*_=−2*w*. For sure, −2*w*≤1−4*w* only if $w\leq \frac{1}{2}$.

### The DCJ-substitution model with distinct operation costs

Now we consider a different model in which substitutions are the content-modifying operations. Recall that substitutions include indels. Again we assign the cost of 1 to each DCJ and the cost of *w*≤1 to each substitution. The *DCJ-substitution distance* of genomes *A* and *B*, denoted by $d_{\text{DCJ}}^{\text{sb}}(A,B)$, is then the minimum cost of a DCJ-substitution sequence that sorts *A* into *B*. If *w*=1, this corresponds exactly to the minimum number of steps required to sort *A* into *B* and can be computed in linear time [8]. Here we present a general method to compute the DCJ-substitution distance for any positive *w*≤1. Similarly to the approach used with the DCJ-indel model, we will first use internal DCJs to obtain a good upper bound and then analyze recombinations to compute the exact DCJ-substitution distance.

#### An upper bound for the DCJ-substitution distance

We can also obtain a good upper bound for the DCJ-substitution distance by showing how to compute the DCJ-substitution distance per component. Given a DCJ operation *ρ*, let *σ*_0_ and *σ*_1_ be, respectively, the sum of the substitution-potentials for the components of the adjacency graph before and after *ρ*, and let *Δσ*(*ρ*)=*σ*_1_−*σ*_0_. If *ρ* is an optimal DCJ internal to a single component of the graph, the definition of substitution-potential implies *Δσ*(*ρ*)≥0. We also have *Δσ*(*ρ*)≥0, if *ρ* is counter-optimal, and *Δσ*(*ρ*)≥−1, if *ρ* is neutral [8]. We define *Δ*_*DCJ*-*σ*_(*ρ*)=*Δ*_*DCJ*_(*ρ*)+*wΔσ*(*ρ*).

After DCJ-sorting a component *P* of *AG*(*A*,*B*), the remaining labels can be easily sorted with substitutions. Let $d_{\text{DCJ}}^{\text{sb}}(P)$ be the DCJ-substitution distance of *P*, that is the minimum cost of a DCJ-substitution sequence of operations sorting *P* separately. This is given by the following proposition.

**Proposition 4. ***For each P*∈*AG*(*A*, *B*), $d_{\text{DCJ}}^{\text{sb}}(P)=d_{\text{DCJ}}(P)+\mathrm{w\sigma}(P)$.

*Proof.* Analogous to the proof of Proposition 3. □

If *P* is a singleton in *AG*(*A*,*B*), $d_{\text{DCJ}}^{\text{sb}}(P)=w$ (the indel of the whole chromosome). A linear cannot be substituted by a circular singleton and *vice-versa*. However, a pair composed by a singleton in genome *A* and a singleton in genome *B*, such that both are linear or both are circular, can be sorted with one substitution (which saves one sorting step per pair). Let *P*_*LS*_ and *P*_*CS*_ be, respectively, the maximum number of disjoint pairs of linear and circular singletons in *AG*(*A*,*B*). Together with Proposition 4, these numbers give a good upper bound for the DCJ-substitution distance:

**Lemma 3.***Given genomes A and B and a positive substitution costw*≤1,

$$d_{\;\text{DCJ}}^{\text{sb}}(A,\;B)\leq d_{\;\text{DCJ}}(A,\;B)+w\underset{P\in A\;G(A,B)}{\sum }\sigma (P)-\;w(P_{L\;S}+P_{C\;S}),$$

where *P*_*LS*_ and *P*_*CS*_ are the numbers of disjoint pairs of linear and circular singletons.

*Proof.* If we sort the components separately we have $d_{\;\text{DCJ}}^{\text{sb}}(A,\;B)\leq \underset{P\in A\;G(A,B)}{\sum }d_{\;\text{DCJ}}^{\text{sb}}(P)$, which, according to Lemma 1 and Proposition 4, corresponds exactly to $d_{\;\text{DCJ}}(A,\;B)+w\underset{P\in A\;G(A,\;B)}{\sum }\sigma (P)$. □

#### Recombinations and the exact DCJ-substitution distance

Now we also need to analyze the effect of path recombinations, that have *Δσ*(*ρ*)≥−2 [8], in the DCJ-substitution distance. Here the space of recombinations is even larger, but can still be efficiently explored. Proposition 2 shows that the substitution-potential increases of one when the number of runs increases of four. Furthermore, when we decrease the number of runs of a path by one, it will decrease the indel-potential only if its initial number of runs is one or a multiple of four. Again, the exact number of runs does not really matter. We have to consider the following properties for each path: 

•whether it is an *AA*, or a *BB*, or an *AB*-path;

•whether it has zero, or a number of runs that is a multiple of four, or a multiple of four plus 1, or a multiple of four plus 2, or a multiple of four plus 3; and

•whether its first run is in *A* or in *B* (by convention, an *AB*-path is always read from *A* to *B*).

**Figure 6:**
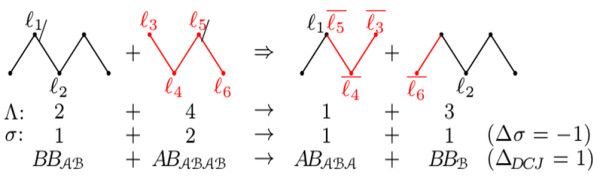
**Neutral recombination that has *Δ***_***DCJ*-*σ***_**=1 −*w* (we represent only the labels of the adjacencies, the cuts of the recombination are represented by “ /”).**

Recall that an empty sequence (with no run) is represented by *ε*. For labeled paths we adopt a different meaning for A**,**B**,**AB**,**BA: for an integer *i*≥0, let A (respectively B) be a sequence with odd 4*i*+1 runs, starting and ending with an A-run (respectively B-run), and let AB (respectively BA), be a sequence with even 4*i*+2 runs, starting with an A-run (respectively B-run) and ending with a B-run (respectively A-run). Here we still have some additional cases: let ABA (respectively BAB) be a sequence with odd 4*i*+3 runs, starting and ending with an A-run (respectively B-run), and let ABAB** (respectively**BABA), be a sequence with even 4*i*+4 runs, starting with an A-run (respectively B-run) and ending with a B-run (respectively A-run). Then, for each type of path (*AA*, *BB* or *AB*) with a particular structure of runs ( A**,**B**,**AB**,**BA**,**ABA, BAB, ABAB**, or**BABA), we have a particular notation. An example of this notation is given in Figure 6, which represents a neutral recombination with *Δ*_*DCJ*-*σ*_=1−*w*.

Again, although each type of recombination can lead to different resultants, it is always possible to choose the “best” resultants in each case: we take the recombination with the smallest *Δ*_*DCJ*-*σ*_, whose resultants can be better reused. In Table 3, we list all recombinations that can have *Δ*_*DCJ*-*σ*_<0, together with those that have *Δ*_*DCJ*-*σ*_=1−*w*≥0, but produce an *AA* or a *BB*-path with runs ABAB or A or B. We denote by ∙ an *AB*-path that never appears as a source in this table (these are all *AB* paths, with the exception of $AB_{ABAB}$ and $AB_{BABA}$).

**Table 3:** Path recombinations that are used to compute the DCJ-substitution distance

	**Sources**	**Result.**	***Δσ***	***Δ***_***DCJ***_	***Δ***_***DCJ-σ***_		**Sources**	**Result.**	***Δσ***	***Δ***_***DCJ***_	***Δ***_***DCJ-σ***_
						*o*_**-1**_	$\;\;AA_{A}+BB_{ABAB}$	$\;∙+AB_{ABAB}$	−1	0	−*w*
*o*_**-2**_	$AA_{ABAB}+BB_{ABAB}$	∙+∙	−2	0	−2*w*	*o*_**-1**_	$\;\;AA_{B}+BB_{ABAB}$	$\;∙+AB_{BABA}$	−1	0	−*w*
						*o*_**-1**_	$\;AA_{ABAB}+BB_{A}$	$\;∙+AB_{BABA}$	−1	0	−*w*
						*o*_**-1**_	$\;AA_{ABAB}+BB_{B}$	$\;∙+AB_{ABAB}$	−1	0	−*w*
*o*_**-1**_	$\;AA_{A}+BB_{ABA}$	∙+∙	−1	0	−*w*						
*o*_**-1**_	$\;AA_{B}+BB_{BAB}$	∙+∙	−1	0	−*w*	*n*_**-2**_	$AA_{ABAB}+AA_{ABAB}$	$\;AA_{ABA}+AA_{BAB}$	−2	1	1−2*w*
*o*_**-1**_	$AA_{ABA}+BB_{A}$	∙+∙	−1	0	−*w*	*n*_**-2**_	$BB_{ABAB}+BB_{ABAB}$	$\;BB_{ABA}+BB_{BAB}$	−2	1	1−2*w*
*o*_**-1**_	$AA_{BAB}+BB_{B}$	∙+∙	−1	0	−*w*	*n*_**-2**_	$AA_{ABAB}+AB_{ABAB}$	$\;∙+AA_{ABA}$	−2	1	1−2*w*
*o*_**-1**_	$\;\;\;\;AA_{AB}+BB_{ABAB}$	∙+∙	−1	0	−*w*	*n*_**-2**_	$AA_{ABAB}+AB_{BABA}$	$\;∙+AA_{BAB}$	−2	1	1−2*w*
*o*_**-1**_	$\;AA_{ABAB}+BB_{AB}$	∙+∙	−1	0	−*w*	*n*_**-2**_	$BB_{ABAB}+AB_{ABAB}$	$\;∙+BB_{BAB}$	−2	1	1−2*w*
*o*_**-1**_	$AA_{AB}+BB_{AB}$	∙+∙	−1	0	−*w*	*n*_**-2**_	$BB_{ABAB}+AB_{BABA}$	$\;∙+BB_{ABA}$	−2	1	1−2*w*
*o*_**-1**_	$\;\;AA_{ABA}+BB_{ABAB}$	∙+∙	−1	0	−*w*	*n*_**-2**_	$AB_{ABAB}+AB_{BABA}$	∙+∙	−2	1	1−2*w*
*o*_**-1**_	$\;\;AA_{BAB}+BB_{ABAB}$	∙+∙	−1	0	−*w*						
*o*_**-1**_	$\;AA_{ABAB}+BB_{ABA}$	∙+∙	−1	0	−*w*	*n*_**-1**_	$\;AA_{A}+AB_{BABA}$	$\;∙+AA_{ABAB}$	−1	1	1−*w*
*o*_**-1**_	$\;AA_{ABAB}+BB_{BAB}$	∙+∙	−1	0	−*w*	*n*_**-1**_	$\;AA_{B}+AB_{ABAB}$	$\;∙+AA_{ABAB}$	−1	1	1−*w*
*o*_**-1**_	$AA_{A}+BB_{A}$	∙+∙	−1	0	−*w*	*n*_**-1**_	$\;BB_{A}+AB_{ABAB}$	$\;∙+BB_{ABAB}$	−1	1	1−*w*
*o*_**-1**_	$AA_{B}+BB_{B}$	∙+∙	−1	0	−*w*	*n*_**-1**_	$\;BB_{B}+AB_{BABA}$	$\;∙+BB_{ABAB}$	−1	1	1−*w*
*o*_**-1**_	$\;\;AA_{A}+BB_{AB}$	∙+∙	−1	0	−*w*	*n*_**-1**_	$\;AA_{AB}+AB_{ABAB}$	$\;∙+AA_{A}$	−1	1	1−*w*
*o*_**-1**_	$\;\;AA_{B}+BB_{AB}$	∙+∙	−1	0	−*w*	*n*_**-1**_	$\;AA_{AB}+AB_{BABA}$	$\;∙+AA_{B}$	−1	1	1−*w*
*o*_**-1**_	$\;AA_{AB}+BB_{A}$	∙+∙	−1	0	−*w*	*n*_**-1**_	$\;BB_{AB}+AB_{BABA}$	$\;∙+BB_{A}$	−1	1	1−*w*
*o*_**-1**_	$\;AA_{AB}+BB_{B}$	∙+∙	−1	0	−*w*	*n*_**-1**_	$\;BB_{AB}+AB_{ABAB}$	$\;∙+BB_{B}$	−1	1	1−*w*

Recombinations of type ***o***_**-*2***_, ***o***_**-*1***_ and ***n***_**-*2***_ can have ***Δ***_***DCJ*-***σ*_***<0***. Recombinations of type ***n***_**-*1***_ have ***Δ***_***DCJ*-*σ***_***≥***0, but produce an ***AA*** or a ***BB***-path with runs ABAB** or**A** or**B.

##### The DCJ-substitution distance formula

In Table 4 we list groups of recombinations, which allow the computation of the exact DCJ-substitution distance, with an approach that greedily maximizes the number of occurrences in U**,**V**,**W**,**$X_{1}$**,**$X_{2}$**,**Y**,**$Z_{1}$** and**$Z_{2}$** in this order. The two groups in**V** are mutually exclusive after maximizing**U**, while those in**W** are subgroups of**V** (they are only computed when there are enough remaining components after maximizing**V). Similarly, each one of the remaining groups are computed when there are enough remaining components after maximizing the upper groups. As previously observed, the recombination is not associative, thus the column ‘DCJ seq.’ determines in which order the sequence of DCJs must be applied in each group. Here we also need to skip some recombinations depending on the value of *w*. In particular, although *Δ*_*DCJ*-*σ*_ indicates that W could be applied for *w*>1/3 and V for *w*>1/4, the last operation of these groups is of type *n*_**-2**_ and increases *Δ*_*DCJ*-*σ*_ for *w*≤1/2. Groups V** and**W are skipped for *w*≤1/2, and their optimal operations are then counted in $X_{1}$.

**Table 4:** All recombination groups that determine the deductions for computing the DCJ-substitution distance

	**Sources**	**Resultants**	**DCJ seq.**	***Δ***_***DCJ*-*σ***_	**skip if**
U	$AA_{ABAB}+BB_{ABAB}$	2 ∙	*o*_**-2**_	−2*w*	
V	$2AA_{ABAB}+BB_{A}+BB_{B}$	4 ∙	2*o*_**-1**_≺*n*_**-2**_	1−4*w*	$w\geq \frac{1}{2}$
	$2BB_{ABAB}+AA_{A}+AA_{B}$	4 ∙		1−4*w*	
W	$AA_{ABAB}+BB_{A}+AB_{ABAB}$	3 ∙	*o*_**-1**_≺*n*_**-2**_	1−3*w*	$w\geq \frac{1}{2}$
	$AA_{ABAB}+BB_{B}+AB_{BABA}$	3 ∙		1−3*w*	
	$BB_{ABAB}+AA_{A}+AB_{BABA}$	3 ∙		1−3*w*	
	$BB_{ABAB}+AA_{B}+AB_{ABAB}$	3 ∙		1−3*w*	
	$2AA_{ABAB}+BB_{A}$	$2\;∙+AA_{BAB}$		1−3*w*	
	$2AA_{ABAB}+BB_{B}$>	$2\;∙+AA_{ABA}$		1−3*w*	
	$2BB_{ABAB}+AA_{A}$	$2\;∙+BB_{BAB}$		1−3*w*	
	$2BB_{ABAB}+AA_{B}$	$2\;∙+BB_{ABA}$		1−3*w*	
$X_{1}$	$AA_{A}+BB_{ABAB}$	$∙+AB_{ABAB}$	*o*_**-1**_	−*w*	
	$AA_{B}+BB_{ABAB}$	$∙+AB_{BABA}$		−*w*	
	$AA_{ABAB}+BB_{A}$	$∙+AB_{BABA}$		−*w*	
	$AA_{ABAB}+BB_{B}$	$∙+AB_{ABAB}$		−*w*	
	$AA_{AB}+BB_{ABAB}$	∙+∙		−*w*	
	$AA_{ABAB}+BB_{AB}$	∙+∙		−*w*	
	$AA_{AB}+BB_{AB}$	∙+∙		−*w*	
	$AA_{ABA}+BB_{ABAB}$	∙+∙		−*w*	
	$AA_{BAB}+BB_{ABAB}$	∙+∙		−*w*	
	$AA_{ABAB}+BB_{ABA}$	∙+∙		−*w*	
	$AA_{ABAB}+BB_{BAB}$	∙+∙		−*w*	
	$AA_{A}+BB_{A}$	∙+∙		−*w*	
	$AA_{B}+BB_{B}$	∙+∙		−*w*	
	$AA_{A}+BB_{AB}$	∙+∙		−*w*	
	$AA_{B}+BB_{AB}$	∙+∙		−*w*	
	$AA_{AB}+BB_{A}$	∙+∙		−*w*	
	$AA_{AB}+BB_{B}$	∙+∙		−*w*	
	$AA_{A}+BB_{ABA}$	∙+∙		−*w*	
	$AA_{B}+BB_{BAB}$	∙+∙		−*w*	
	$AA_{ABA}+BB_{A}$	∙+∙		−*w*	
	$AA_{BAB}+BB_{B}$	∙+∙		−*w*	
$X_{2}$	$AA_{ABAB}+AA_{ABAB}$	$AA_{ABA}+AA_{BAB}$	*n*_**-2**_	1−2*w*	$w\geq \frac{1}{2}$
	$BB_{ABAB}+BB_{ABAB}$	$BB_{ABA}+BB_{BAB}$		1−2*w*	
	$AA_{ABAB}+AB_{ABAB}$	$∙+AA_{ABA}$		1−2*w*	
	$AA_{ABAB}+AB_{BABA}$	$∙+AA_{BAB}$		1−2*w*	
	$BB_{ABAB}+AB_{ABAB}$	$∙+BB_{BAB}$		1−2*w*	
	$BB_{ABAB}+AB_{BABA}$	$∙+BB_{ABA}$		1−2*w*	
	$AB_{ABAB}+AB_{BABA}$	∙+∙		1−2*w*	
Y	$2AB_{ABAB}+AA_{B}+BB_{A}$	4 ∙	2*n*_**-1**_≺*o*_**-2**_	2−4*w*	$w\geq \frac{1}{2}$
	$2AB_{BABA}+AA_{A}+BB_{B}$	4 ∙		2−4*w*	
$Z_{1}$	$AB_{ABAB}+AA_{AB}+BB_{ABA}$	3 ∙	*n*_**-1**_≺*o*_**-1**_	1−2*w*	$w\geq \frac{1}{2}$
	$AB_{BABA}+AA_{AB}+BB_{BAB}$	3 ∙		1−2*w*	
	$AB_{BABA}+AA_{ABA}+BB_{AB}$	3 ∙		1−2*w*	
	$AB_{ABAB}+AA_{BAB}+BB_{AB}$	3 ∙		1−2*w*	
	$AB_{ABAB}+AA_{B}+BB_{ABA}$	3 ∙		1−2*w*	
	$AB_{ABAB}+AA_{BAB}+BB_{A}$	3 ∙		1−2*w*	
	$AB_{BABA}+AA_{A}+BB_{BAB}$	3 ∙		1−2*w*	
	$AB_{BABA}+AA_{ABA}+BB_{B}$	3 ∙		1−2*w*	
	$AB_{ABAB}+AA_{B}+BB_{A}$	$2\;∙+AB_{BABA}$		1−2*w*	
	$AB_{BABA}+AA_{A}+BB_{B}$	$2\;∙+AB_{ABAB}$		1−2*w*	
$Z_{2}$	$2AB_{ABAB}+AA_{B}$	$2\;∙+AA_{ABA}$	*n*_**-1**_≺*n*_**-2**_	2−3*w*	$w\geq \frac{2}{3}$
	$2AB_{ABAB}+BB_{A}$	$2\;∙+BB_{BAB}$		2−3*w*	
	$2AB_{BABA}+AA_{A}$	$2\;∙+AA_{BAB}$		2−3*w*	
	$2AB_{BABA}+BB_{B}$	$2\;∙+BB_{ABA}$		2−3*w*	

The recombinations allow us to obtain an exact formula for the DCJ-substitution distance:

###### Theorem 3

Given genomes A and B and a positive substitution cost *w*≤1,

$$\begin{array}{lll} d_{\text{DCJ}}^{\text{sb}}(A,\;B) & =d_{\;\text{DCJ}}(A,\;B)+w\underset{P\in A\;G(A,\;B)}{\sum }\sigma (P)\; & \;\\ & \;-\;2wU-(4w-1)V-(3w-1)W\; & \;\\ & \;-\;wX_{1}-(2w-1)(X_{2}+2Y+Z_{1})\; & \;\\ & \;-\;(3w-2)Z_{2}-\;w(P_{L\;S}+P_{C\;S}),\; & \; \end{array}$$

*where*U**,**V**,**W**,**$X_{1}$**,**$X_{2}$**,**Y**,**$Z_{1}$*and*$Z_{2}$*are computed as described above andP*_*LS*_ and *P*_*CS *_*are the numbers of disjoint pairs of linear and circular singletons.*

Observe that the number of occurrences in each group depends only on *w* and the initial number of each type of component and, for any 2/3<*w*≤1, our formula is equivalent to the one proposed in [8], since the same occurrences of groups of recombinations and an equivalent upper bound are taken into account.

### Complexity

Both *AG*(*A*,*B*) and *d*_*DCJ*_(*A*,*B*) can be computed in linear time [3]. The occurrences in each recombination group depends only on *w* and the initial components. The runs are obtained by a single walk through each path, thus the whole procedure takes linear time for both models.

### Establishing the triangular inequality

We have presented two genomic distances that combine DCJ and content-modifying operations and can be computed in linear time. However, content-modifying operations are applied to pieces of DNA of any size, and a side effect of this fact is that the triangular inequality often does not hold for distances that consider these operations [4,6]**-**[8,12].

Let *A*, *B* and *C* be three genomes, with unequal contents, and consider, without loss of generality, that $d_{\text{DCJ}}^{\text{id}}(A,B)\geq d_{\text{DCJ}}^{\text{id}}(A,C)$ and $d_{\text{DCJ}}^{\text{id}}(A,B)\geq d_{\text{DCJ}}^{\text{id}}(B,C)$. The triangular inequality is then the property which guarantees that the inequality $d_{\text{DCJ}}^{\text{id}}(A,B)\leq d_{\text{DCJ}}^{\text{id}}(A,C)+d_{\text{DCJ}}^{\text{id}}(B,C)$also holds. Unfortunately this is not the case for the DCJ-indel distance, and also not the case for the DCJ-substitution distance. Take for example the genomes *A*={∘*abcde*∘}, $B=\{\circ \text{ac}\bar{d}\text{be}\circ \}$ and *C*={∘*ae*∘} [6]. While the cost of sorting *A* (or *B*) into *C* is *w* (one indel), the minimum number of DCJs (that are inversions in this case) required to sort *A* into *B* is three. We have $d_{\text{DCJ}}^{\text{id}}(A,B)=3$**,**$d_{\text{DCJ}}^{\text{id}}(A,C)=w$, $d_{\text{DCJ}}^{\text{id}}(B,C)=w$ and the triangular inequality is disrupted.

**Figure 7:**
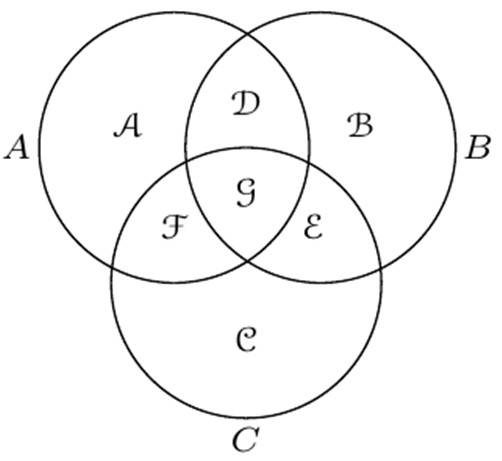
The set of markers of each genome is represented by a circle.

Denote by A**,**B**,**C**,**D**,**E**,**F and G the disjoint sets of markers such that: A**,**B or C are the sets of markers that occur respectively only in genome *A*, *B* or *C*, the markers in D are common only to genomes *A* and *B*, the markers in E are common only to *B* and *C*, the markers in F are common only to *A* and *C*, and, Gis the set of markers that are common to all three genomes *A*, *B* and *C*. These sets are represented in Figure 7.

When D=∅, meaning that genomes *A* and *B* have no common marker that does not occur in *C*, the triangular inequality holds for both DCJ-indel and DCJ-substitution distances [12]. However, if D≠∅, the triangular inequality can be disrupted for $d_{\;\text{DCJ}}^{\text{id}}$ and $d_{\;\text{DCJ}}^{\text{sb}}$, and this may be an obstacle if one intends to use these distances to compute the median of three or more genomes and in phylogenetic reconstructions.

It is possible to establish the triangular inequality in our two models *a posteriori*, by adapting an approach proposed in [12]: we simply sum to each distance a surcharge that depends on the number of unique markers, as we will see in the following subsections.

We define the diameter as the maximum distance between any pair of genomes, usually as a function on the size of the genomes. We use this definition in the next results.

#### Correction for the DCJ-indel distance

For genomes *A* and *B* and a positive constant *k*, let $m^{\text{id}}(A,B)=d_{\text{DCJ}}^{\text{id}}(A,B)+k\cdot u(A,B)$, where *u*(*A*,*B*) is the number of unique markers between *A* and *B*[7]**,**[12]. We then have $m^{\text{id}}(A,B)=d_{\text{DCJ}}^{\text{id}}(A,B)+k(|A|+|F|+|B|+|E|)$**,**$m^{\text{id}}(A,C)=d_{\text{DCJ}}^{\text{id}}(A,C)+k(|A|+|D|+|C|+|E|)$ and $m^{\text{id}}(B,C)=d_{\text{DCJ}}^{\text{id}}(B,C)+k(|B|+|D|+|C|+|F|)$. From this definition we can derive a simpler inequality that can be used to determine the value of the constant *k*:

**Proposition 5** (from [12]). Given three genomes *A*, *B* and *C* without duplicated markers, the inequality *m*^*id*^(*A*,*B*)≤*m*^*id*^(*A*,*C*)+*m*^*id*^(*B*,*C*) holds if, and only if, $d_{\text{DCJ}}^{\text{id}}(A,B)\leq d_{\text{DCJ}}^{\text{id}}(A,C)+d_{\text{DCJ}}^{\text{id}}(B,C)+2k|D|$*, where*D*is the set of markers common only toA* and *B*.

The problem now is to find the minimum value of *k* for which the inequality of Proposition 5 holds. In order to accomplish this task, the first step is to determine the diameter of the DCJ-indel distance.

**Lemma 4. ***Given a positive indel cost w*≤1 *and two genomes A and B with n common markers, then*

$$d_{\text{DCJ}}^{\text{id}}(A,B)\leq (w+1)n+w(L_{A}+S_{A}+L_{B}+S_{B}),$$

where *L*_*A*_, *S*_*A*_ and *L*_*B*_, *S*_*B*_* are, respectively, the number of linear chromosomes and circular singletons in genomesA* and *B*.

*Proof.* Let |*P*| be the number of vertices in component *P*, that is DCJ-sorted with $\left\lfloor \frac{|P|-\;1}{2}\right\rfloor$DCJs [14]. If |*P*| is even, *P* is sorted with $\frac{\left|P\right|}{2}\;-\;1$ DCJs and $\lambda (P)\leq \frac{\left|P\right|}{2}\;+\;1$ indels, then $d_{\;\text{DCJ}}^{\text{id}}(P)\leq \frac{\left|P\right|}{2}\;-\;1\;+\;w(\frac{\left|P\right|}{2}\;+\;1)=\frac{\left(w\;+\;1)|P\right|}{2}\;+\;w\;-\;1$. If |*P*| is odd, *P* is sorted with $\frac{|P|\;-\;1}{2}$ DCJs and $\lambda (P)\leq \frac{|P|\;+\;1}{2}$ indels, then $d_{\;\text{DCJ}}^{\text{id}}(P)\leq \frac{|P|\;-\;1}{2}\;+\;w\frac{|P|\;+\;1}{2}=\frac{(w\;+\;1)|P|\;+\;w\;-\;1}{2}$. As *w*≤1 implies $w\;-\;1\leq \frac{w\;-\;1}{2}\leq 0$, for any component *P* we have $d_{\;\text{DCJ}}^{\text{id}}(P)\leq \frac{(w\;+\;1)|P|\;+\;w\;-\;1}{2}$. Then, $d_{\;\text{DCJ}}^{\text{id}}(A,\;B)\leq \underset{P\in A\;G(A,B)}{\sum }\;d_{\;\text{DCJ}}^{\text{id}}(P)\leq \underset{P\in A\;G(A,B)}{\sum }\;\;\frac{(w\;+\;1)|P|\;+\;w\;-\;1}{2}=\frac{w\;+\;1}{2}\underset{P\in A\;G(A,B)}{\sum }\;\;|P|+\underset{P\in A\;G(A,B)}{\sum }\;\;\frac{w-1}{2}$. Each linear chromosome corresponds to one path in *AG*(*A*, *B*), thus the number of components is at least (*L*_*A*_ + *S*_*A*_ + *L*_*B*_ + *S*_*B*_) and $\underset{P\in A\;G(A,B)}{\sum }\;\;\frac{w-1}{2}\leq \frac{\left(L_{A}\;+\;S_{A}\;+\;L_{B}\;+\;S_{B})(w-1\right)}{2}\leq 0$. Furthermore, from [12] we know that $\underset{P\in A\;G(A,B)}{\sum }|P|=2n\;+\;L_{A}\;+\;S_{A}\;+\;L_{B}\;+\;S_{B}$. □

We are ready to generalize the result of [12], and determine the minimum possible value of *k*.

**Theorem 4.** For any positive indel cost *w*≤1, the function *m*^*id*^* satisfies the triangular inequality if and only if*$k\geq \frac{w+1}{2}$.

*Proof.* Recall that, to prove the triangular inequality for *m*^*id*^, we only need to find a *k* such that $d_{\;\text{DCJ}}^{\text{id}}(A,\;B)\leq d_{\;\text{DCJ}}^{\text{id}}(A,C)+d_{\;\text{DCJ}}^{\text{id}}(B,\;C)+2k|D|$ holds (Proposition 5). We know that the inequality holds when D=∅[12]. It remains to examine the case in which D≠∅. The worst case would be to have an empty genome *C*[12]. Let *X*_*A*_ and *X*_*B*_ be the number of chromosomes in *A* and *B*. Since *C* is empty, we know that $d_{\;\text{DCJ}}^{\text{id}}(A,\;C)=wX_{A}$ and $d_{\;\text{DCJ}}^{\text{id}}(B,\;C)=wX_{B}$. From Lemma 4, we have $d_{\;\text{DCJ}}^{\text{id}}(A,\;B)\leq (w\;+\;1)|D|\;+\;w(L_{A}\;+\;S_{A}\;+\;L_{B}\;+\;S_{B})$. This gives $\left(w\;+\;1)|D|\;+\;w(L_{A}\;+\;S_{A}\;+\;L_{B}\;+\;S_{B})\leq w(X_{A}\;+\;X_{B})\;+\;2k|D\right|$. Since *L*_*A*_ + *S*_*A*_ + *L*_*B*_ + *S*_*B*_≤*X*_*A*_ + *X*_*B*_, we have (w+1)|D|≤2k|D|, which holds for any $k\geq \frac{w\;+\;1}{2}$.

For the necessity, take *A* and *B* with *n* common markers and let each genome be composed of one circular chromosome, meaning that we have one adjacency per common marker in each genome (or *n* adjacencies per genome). Then let *AG*(*A*, *B*) have one single cycle with 2*n* vertices and let each vertex be labeled, so that the number of runs in the cycle is 2*n* and the number of unique markers in each genome is *n*. Thus, we have $d_{\;\text{DCJ}}^{\text{id}}(A,\;B)=(n\;-\;1)\;+\;w(n\;+\;1)=(w\;+\;1)n\;+\;(w\;-\;1)$ and the corrected distance is *m*^*id*^(*A*, *B*)=(*w* + 1)*n* + (*w* − 1) + 2*kn*. Take *C* as an empty genome, so that $d_{\;\text{DCJ}}^{\text{id}}(A,\;C)=d_{\;\text{DCJ}}^{\text{id}}(B,\;C)=w$ and *m*^*id*^(*A*, *C*)=*m*^*id*^(*B*, *C*)=*w* + 2*kn*. The inequality *m*^*id*^(*A*, *B*)≤*m*^*id*^(*A*, *C*) + *m*^*id*^(*B*, *C*) corresponds to (*w* + 1)*n* + (*w* − 1) + 2*kn*≤2*w* + 4*kn* or, equivalently, 2*kn*≥(*w* + 1)*n* − *w* − 1, that is $k\geq \frac{w+1}{2}\left(1-\frac{1}{n}\right)$, which holds for all *n* only if $k\geq \frac{w+1}{2}$. □

## Correction for the DCJ-substitution distance

Similarly, in the case of the DCJ-substitution distance, for genomes *A* and *B* and a positive constant *k*^**′**^, let $m^{\text{sb}}(A,\;B)=d_{\;\text{DCJ}}^{\text{sb}}(A,\;B)+k^{\prime }\cdot u(A,\;B)$, where *u*(*A*, *B*) is the number of unique markers between *A* and *B*[7]**,**[12]. We then have $m^{\text{sb}}(A,\;B)=d_{\;\text{DCJ}}^{\text{sb}}(A,\;B)\;+\;k^{\prime }(|A|\;+\;|F|\;+\;|B|\;+\;|E|)$**,**$m^{\text{sb}}(A,\;C)=d_{\;\text{DCJ}}^{\text{sb}}(A,\;C)\;+\;k^{\prime }(|A|\;+\;|D|\;+\;|C|\;+\;|E|)$ and $m^{\text{sb}}(B,\;C)=d_{\;\text{DCJ}}^{\text{sb}}(B,\;C)\;+\;k^{\prime }(|B|\;+\;|D|\;+\;|C|\;+\;|F|)$. Again, from this definition we can derive a simpler inequality that can be used to determine the value of the constant *k*^**′**^:

**Proposition 6** (from [12]). *Given three genomes A, B and C without duplicated markers, the inequality m*^*sb*^(*A*, *B*)≤*m*^*sb*^(*A*, *C*)+*m*^*sb*^(*B*, *C) holds if, and only if,*$d_{\;\text{DCJ}}^{\text{sb}}(A,\;B)\leq d_{\;\text{DCJ}}^{\text{sb}}(A,\;C)+d_{\;\text{DCJ}}^{\text{sb}}(B,\;C)+2k^{\prime }|D|$, where D is the set of markers common only to *A* and *B*.

In order to find the minimum value of *k*^**′**^ for which the inequality of Proposition 6 holds, we need to determine the diameter of the DCJ-substitution distance, that is given by the following lemma.

**Lemma 5. ***If A and B are genomes with n common markers, then*

$$d_{\;\text{DCJ}}^{\text{sb}}(A,\;B)\leq \frac{\left(w+2\right)}{2}n+w(L_{A}+S_{A}+L_{B}+S_{B}),$$

where *L*_*A*_, *S*_*A*_, *L*_*B*_ and *S*_*B*_ are, respectively, the number of linear chromosomes and circular singletons in genomes *A* and *B*.

*Proof.* Let |*P*| be the number of vertices in component *P*, that is DCJ-sorted with $\left\lfloor \frac{|P|\;-\;1}{2}\right\rfloor$ DCJs [14]. If |*P*| is even, then *P* can be DCJ-sorted with $\frac{\left|P\right|}{2}-1$ DCJs. We have to analyze two cases: (i) if |*P*|=4*x*+4, then $\sigma (P)\leq \frac{\left|P\right|}{4}+1$ and $d_{\;\text{DCJ}}^{\text{sb}}(P)\leq (\frac{\left|P\right|}{2}-1)+w(\frac{\left|P\right|}{4}+1)=\frac{\left(w+2)|P\right|}{4}+w-1$; (ii) if |*P*|=4*x*+2, then $\sigma (P)\leq \frac{|P|-2}{4}+1$ and $d_{\;\text{DCJ}}^{\text{sb}}(P)\leq (\frac{\left|P\right|}{2}-1)+w(\frac{|P|-2}{4}+1)=\frac{\left(w+2)|P\right|}{4}+\frac{w-2}{2}$. As *w*≤1 implies $\frac{w-2}{2}\leq w-1\leq 0$. If |*P*| is odd, then *P* is an *AA*- or a *BB*-path and can be DCJ-sorted with $\frac{|P|-1}{2}$ DCJs. Again, we have to analyze two cases: (i) if |*P*|=4*x*+3, then $\sigma (P)\leq \frac{|P|+1}{4}$ and $d_{\;\text{DCJ}}^{\text{sb}}(P)\leq \frac{|P|-1}{2}+w(\frac{|P|+1}{4})=\frac{\left(w+2)|P\right|}{4}+\frac{w-2}{4}$; (ii) if |*P*|=4*x+1, then*$\sigma (P)\leq \frac{|P|+3}{4}$ and $d_{\;\text{DCJ}}^{\text{sb}}(P)\leq \frac{|P|-1}{2}+w(\frac{|P|+3}{4})=\frac{\left(w+2)|P\right|}{4}+\frac{3w-2}{4}$. In this last case we could have $d_{\;\text{DCJ}}^{\text{sb}}(P)>\frac{\left(w+2)|P\right|}{4}$. Observe however that the numbers of *AA*- and *BB*-paths are bounded, respectively, by *L*_*A*_ and *L*_*B*_. Then, $d_{\;\text{DCJ}}^{\text{sb}}(A,B)\leq \underset{P\in A\;G(A,B)}{\sum }\;d_{\;\text{DCJ}}^{\text{sb}}(P)\leq \underset{P\in A\;G(A,B)}{\sum }\;\;\frac{\left(w\;+\;2)|P\right|}{4}+\frac{\left(3w-2)(L_{A}+L_{B}\right)}{4}=\frac{w\;+\;2}{4}\underset{P\in A\;G(A,B)}{\sum }\;\;|P|+\frac{\left(3w-2)(L_{A}+L_{B}\right)}{4}$. From [12] we know that $\underset{P\in A\;G(A,B)}{\sum }|P|=2n\;+\;L_{A}\;+\;S_{A}\;+\;L_{B}\;+\;S_{B}$. Therefore, $d_{\;\text{DCJ}}^{\text{sb}}(A,\;B)\leq \frac{w\;+\;2}{4}(2n\;+\;L_{A}\;+\;S_{A}\;+\;L_{B}\;+\;S_{B})+\frac{\left(3w-2)(L_{A}+L_{B}\right)}{4}=\frac{\left(w+2\right)}{2}n+w(L_{A}+L_{B})+\frac{\left(w+2)(S_{A}+S_{B}\right)}{4}\leq \frac{\left(w+2\right)}{2}n+w(L_{A}+L_{B}+S_{A}+S_{B})$. □

We are ready to generalize the result of [12], and determine the minimum possible value of*k*^**′**^.

**Theorem 5.** For any positive substitution cost *w*≤1, the function *m*^*sb*^* satisfies the triangular inequality if and only if*$k^{\prime }\geq \frac{w+2}{4}$.

*Proof.* Recall that, to prove the triangular inequality for *m*^*sb*^, we only need to find a *k*^**′**^ such that $d_{\;\text{DCJ}}^{\text{sb}}(A,\;B)\leq d_{\;\text{DCJ}}^{\text{sb}}(A,\;C)+d_{\;\text{DCJ}}^{\text{sb}}(B,\;C)+2k^{\prime }|D|$ holds (Proposition 6). We know that the inequality holds when D=∅[12]. It remains to examine the case in which D≠∅. The worst case would be to have an empty genome *C*[12]. Let *X*_*A*_ and *X*_*B*_ be the number of chromosomes in *A* and *B*. Since *C* is empty, we know that $d_{\;\text{DCJ}}^{\text{sb}}(A,\;C)=wX_{A}$ and $d_{\;\text{DCJ}}^{\text{sb}}(B,\;C)=wX_{B}$. From Lemma 5, we have $d_{\;\text{DCJ}}^{\text{sb}}(A,\;B)\leq \frac{\left(w\;+\;2)|D\right|}{2}\;+\;w(L_{A}\;+\;S_{A}\;+\;L_{B}\;+\;S_{B})$. This gives $\frac{\left(w\;+\;2)|D\right|}{2}\;+\;w(L_{A}\;+\;S_{A}\;+\;L_{B}\;+\;S_{B})\leq w(X_{A}\;+\;X_{B})\;+\;2k^{\prime }|D|$. Since *L*_*A*_ + *S*_*A*_ + *L*_*B*_ + *S*_*B*_≤*X*_*A*_ + *X*_*B*_, we have $\frac{\left(w\;+\;2)|D\right|}{2}\leq 2k^{\prime }|D|$, which holds for any $k^{\prime }\geq \frac{w\;+\;2}{4}$.

For the necessity, take *A* and *B* with *n* common markers, for *n* even, and let each genome be composed of one circular chromosome, meaning that we have one adjacency per common marker in each genome (or *n* adjacencies per genome). Then let *AG*(*A*, *B*) have one single cycle with 2*n* vertices and let each vertex be labeled, so that the number of runs in the cycle is 2*n* and the number of unique markers in each genome is *n*. Thus, we have $d_{\;\text{DCJ}}^{\text{sb}}(A,\;B)=(n\;-\;1)\;+\;w(\frac{n}{2}\;+\;1)=\frac{(w+2)n}{2}\;+\;(w\;-\;1)$ and the corrected distance is $m^{\text{sb}}(A,\;B)=\frac{(w+2)n}{2}\;+\;(w\;-\;1)\;+\;2k^{\prime }n$. Take *C* as an empty genome, so that $d_{\;\text{DCJ}}^{\text{sb}}(A,\;C)=d_{\;\text{DCJ}}^{\text{sb}}(B,\;C)=w$ and *m*^*sb*^(*A*, *C*)=*m*^*sb*^(*B*, *C*)=*w* + 2*k*^**′**^*n*. The inequality *m*^*sb*^(*A*, *B*)≤*m*^*sb*^(*A*, *C*) + *m*^*sb*^(*B*, *C*) corresponds to $\frac{(w+2)n}{2}\;+\;(w\;-\;1)\;+\;2k^{\prime }n\leq 2w\;+\;4k^{\prime }n$ or, equivalently, $2k^{\prime }n\geq (\frac{w\;+\;2}{2})n\;-\;w\;-\;1$, that is $k^{\prime }\geq \frac{w+2}{4}\;-\;\frac{w+1}{2n}$, which holds for all *n* only if $k^{\prime }\geq \frac{w+2}{4}$. □

## Conclusions

In this work we have presented methods to compute in linear time the DCJ-indel and DCJ-substitution distances between two genomes without duplicated markers, when the content-modifying cost is distinct from and upper bounded by the DCJ cost. Content-modifying operations can be applied to pieces of DNA of any size, and a side effect of this property is that the triangular inequality does not hold for our distance formulas. However we have shown that an *a posteriori* correction can be applied to establish the triangular inequality in both DCJ-indel and DCJ-substitution distances.

## Competing interests

The authors declare that they have no competing interests.

## Authors’ contributions

PHS, RM, SD and MDVB have elaborated the model, proved the results and written the paper. All authors read and approved the final manuscript.
